# Apples and oranges: Conceptual review as task analysis method^1^

**DOI:** 10.1111/ejn.16623

**Published:** 2025-01-13

**Authors:** Annemarie van Stee

**Affiliations:** ^1^ Department of Philosophy Radboud University Nijmegen The Netherlands

**Keywords:** cognitive ontology, meta‐analysis, philosophy, self‐reflection, tasks, validity

## Abstract

Conceptual review is a method to address issues of task comparability and task validity in cognitive neuroscience. Meta‐analyses within cognitive neuroscience (CNS) as well as integration of neuroscientific findings with findings from adjacent disciplines both involve gathering studies that have purportedly investigated the same mental concept. After all, it is no use comparing apples and oranges. Tasks, and in particular the experimental contrasts implemented through tasks, determine whether studies are in fact comparable. Yet studies tend to be grouped together or kept apart based on the mental label researchers have applied and unfortunately, labels are an unreliable proxy for experimental contrasts. Different contrasts may receive the same label: ‘working memory’ studies rely on a variety of contrasts, derived from a variety of tasks. Vice versa, the same contrast may receive different labels: ‘task switching’ and ‘working memory’ studies can be exactly the same in terms of their experimental contrast. Label use thus obscures comparability problems. What is more, even when experimental contrasts are comparable, they may be invalid operationalizations of the mental label attached to them. In this paper, I introduce conceptual review as a method for task analysis. It can stand on its own or be combined with a cognitive ontology. Conceptual review applies philosophical strategies for analysing concepts to methodological choices in CNS studies, to uncover their conceptual implications. Conceptual review thus sheds light on the precise concept that was studied and thereby, on the comparability of CNS studies and the validity of tasks.

## Graphical abstract

Conceptual review is a method to analyse tasks in cognitive neuroscience (CNS), to determine what mental label (e.g. ‘working memory’ or ‘self‐reflection’) would be appropriate. This helps to:

- ensure the validity of tasks and the proper communication of the scope of findings;
- shed light on the comparability of studies that are included in meta‐analyses within CNS or across disciplines;
- improve cognitive ontologies through basing a taxonomy on facts about tasks.

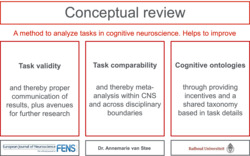

**Abbreviations**
CNS: cognitive neuroscience

## 1 TASK COMPARABILITY AND TASK VALIDITY

The number of neuroimaging studies has grown exponentially over the past decades. To be able to see the overall patterns in the results of all these studies, large‐scale meta‐analysis is necessary. Online databases, such as Neurosynth (neurosynth.org) or BrainMap (brainmap.org), employ informatics to enable large‐scale meta‐analyses of neuroimaging studies. Naturally, meta‐analyses should only compare studies that are in fact comparable. In practice, however, this requirement can be tricky and is often not met (see e.g. Borenstein et al., 2021).

Databases currently annotate imaging data with *labels* for mental processes. Researchers enter these labels manually (BrainMap) or they are extracted automatically from research articles (Neurosynth). Other types of meta‐analysis, e.g. in review articles, also tend to select and group studies on the basis of the mental labels researchers have applied. In such cases, labels are a proxy for the tasks used to obtain data, or more specifically, for the experimental contrast implemented through a task. Experimental contrasts determine what mental processes are involved and are thereby the crucial factor for comparability between studies. Yet labels may be applied inconsistently to contrasts, for example when two different contrasts receive the same mental label or, vice versa, when the same contrast is employed in different CNS subfields and labelled differently accordingly (Figdor, 2011; Francken & Slors, 2014; Poldrack et al., 2011; Poldrack & Yarkoni, 2016; Sullivan, 2009).

For example, ‘working memory’ is a mental label applied to neuroimaging studies employing the N‐back task, but also to studies employing the Wisconsin card‐sorting task, or the Sternberg task, or others. What is more, the Sternberg task allows for different experimental contrasts, such as high load versus low load, or probe match versus probe mismatch (Poldrack et al., 2011). An automated meta‐analysis on ‘working memory’ will thus include a wide range of different experimental contrasts. Vice versa, the Wisconsin card‐sorting task is not just used in ‘working memory’ studies, but also in ‘task‐switching’ studies (Francken & Slors, 2014). Given these different labels, meta‐analyses will not group together Wisconsin card‐sorting studies, even when they are entirely comparable in terms of their experimental contrasts. In other words, we are comparing apples and oranges and failing to compare apples with apples. I call this the *comparability problem*.

What is more, even where consensus is to be had, it may reflect mistaken assumptions of the research community. For example, it has been questioned whether the N‐back task, though widely used as a working memory task, is a valid operationalization of working memory at all (Jaeggi et al., 2010; Kane et al., 2007; Miller et al., 2009; Redick & Lindsey, 2013). In the broad terms that are most relevant to neuroscience researchers, a task is valid when it measures what it purports to be measuring (Borsboom et al., 2004; T. L. Kelley, 1927). *Problems of validity* arise when tasks do not measure or induce the mental process they purportedly investigate.^2^ If it is true that the N‐back task does not operationalize working memory, then studies employing it are invalid investigations of working memory and its neural correlates. Whatever such studies may be investigating, ‘working memory’ is an inappropriate label.

Problems of validity and comparability do not just hinder progress in cognitive neuroscience itself, they also hinder collaboration across disciplines. Explanations from cognitive psychology and cognitive neuroscience, for example, can only be integrated if the cognitive labels they have in common refer to the same cognitive processes. Unfortunately, this is often not the case, as the processes are induced by tasks that sometimes differ radically from one another, both within and between disciplines (Sullivan, 2016a, 2016b).

Many of the authors noting the widespread problems of validity and/or comparability in cognitive neuroscience think of cognitive ontologies as (part of) a solution (Figdor, 2011; Francken & Slors, 2014; Poldrack et al., 2011; Poldrack & Yarkoni, 2016; Sullivan, 2016a, 2017). Cognitive ontologies attempt to standardize terminology regarding cognition, to facilitate mutual understanding between researchers and automated aggregation of research findings (Janssen et al., 2017; Poldrack et al., 2011). Some authors remain neutral on the question of how such cognitive ontologies should be built up; one could either start from the mental concepts (‘working memory’) and their definitions or from tasks, identifying the latter by names such as Sternberg task (e.g. Francken & Slors, 2014). The best‐known attempt at a cognitive ontology, Cognitive Atlas (cognitiveatlas.org; Poldrack et al., 2011; Poldrack & Yarkoni, 2016), takes its starting point in mental concepts and their definitions.^3^ Tasks are not specified through factual details about stimuli and instructions, but also through definitions.^4^ The problem with this strategy is that all such definitions are contested. Cognitive Atlas offers wiki‐style discussion pages to resolve disagreements and where they cannot be resolved, the option of ‘forking’ a concept.^5^ In this way, however, Cognitive Atlas does not solve the comparability problem but merely shows that it exists.

In contrast to neutrality or mental concepts first, I would argue that for a cognitive ontology to be fruitful, it should be built from the task details up. With Carrie Figdor, I would argue that “a useful cognitive ontology for cognitive neuroscience depends essentially on a useful task ontology” (2011, p. 224). Furthermore, I would argue that relying on names for tasks is not enough, as applying them in different ways can lead to different experimental contrasts, as in the Sternberg task example mentioned above. The principled advantage of starting with the task details is that those determine the experimental contrast and delineate the scope of the study. The practical advantage of working from such methodological details up is that it is impossible for researchers to disagree about what stimuli were used in a particular study, or what instructions were given, whereas when you start with mental concepts, you are in disputed terrain from the start. Beyond what Figdor or any other authors have proposed, I show in the next section how building up a task ontology can be done exactly, and how analysis of tasks can lead to an explication of the mental concepts they operationalize.

In general, then, labels are currently too often unreliable indicators of what has been studied exactly and how that compares to what other researchers have studied under that label. Unfortunately, such “conceptual challenges […] remain widely underappreciated within the neuroimaging community” (Poldrack & Yarkoni, 2016). Task analysis is needed to ensure labels are valid and studies are comparable to one another. In the next section, I introduce conceptual review as a method for task analysis. It applies philosophical strategies for analysing conceptual assumptions to tasks used in empirical research. After outlining the steps involved in conceptual review, I also discuss how it compares to what neuroscientists already do, e.g. checking for confounds or testing for convergent validity. In section three, I discuss the implementation of conceptual review and how to facilitate the uptake of its results by the wider CNS community.

## 2 CONCEPTUAL REVIEW

I developed conceptual review as a method to critically analyse tasks for their conceptual implications (van Stee, 2022). Conceptual review results in a precise understanding of the mental concept that has been studied. This is important in itself, to ensure the validity of tasks and the proper communication of the scope of findings. It is also a crucial step in ensuring that when scientific findings are integrated with one another, they are offering perspectives on the same thing, rather than comparing apples and oranges.

Conceptual review is a type of review, as it pertains to an entire CNS literature, just like ordinary review articles in CNS do. Sometimes it may be possible to review all studies in a CNS field, as in the example of CNS of self‐reflection below. At other times, the number of studies may be too large and conceptual review needs to be performed on a subset only (e.g. the most influential studies in the field as determined by number of citations). Time investment for conceptual review is similar to ordinary review. Yet whereas ordinary review articles in CNS aim to uncover patterns in the neural data found in a large number of studies, conceptual review does not focus on the neural side of CNS but on explicating what mental concept has been studied exactly. Ordinary reviews and automated forms of meta‐analysis can make use of this information to ensure that they compare studies that are in fact comparable. The results of the conceptual review can also be used to understand how studies that use different tasks relate to each other. Where necessary, task choices can be critiqued and alternatives suggested.

Conceptual review is called *conceptual* review because it analyses the conceptual implications of tasks. It shares some strategies with how philosophers analyse concepts; I will describe these strategies in section 2.2. When philosophers analyse concepts, they aim to explicate the conditions under which a concept applies. Ideally, there is only one set of such conditions; philosophers aim to uncover *the* way in which a concept applies. Conceptual review, on the other hand, takes its starting point in the methods CNS researchers have used. It teases out the conceptual implications of these methods. Insofar as several tasks have been developed, conceptual review is bound to uncover *several* conceptualizations.

Conceptual review could be performed by neuroscientists who have some background in philosophy, or by philosophers who know how to read the methods sections of CNS articles, or by philosopher‐neuroscientist teams. Most neuroscientists and philosophers will not be inclined to do conceptual reviews themselves and that is not a problem; just like we do not need all neuroscientists to perform ordinary reviews, it will be enough if only some people do conceptual reviews. Table 1 provides an outline of the conceptual review process. In this section, I explain how conceptual review proceeds and illustrate its steps with a conceptual review of CNS of self‐reflection.

**TABLE 1:** Outline of conceptual review.

**Phase 1. Setting up an inventory**	*Mental label used*
	*Stimuli* incl. presentation times
	*Instructions* incl. required response, allotted response time
	*Control condition*
	*Other details that influence mental concept studied* e.g. post‐scan questionnaires, participant characteristics
**Phase 2.** **Analysing conceptual implications** Possible strategies:	*Comparing studies to each other* What methodological choices do many/most studies share? What differences between studies in the inventory strike you? Can groups of methodological approaches be distinguished? What precise mental concepts do these approaches capture?
	*Investigating the history of a methodological approach* In what context and with what aims were the task and stimuli developed? What aspects of the methods are explained by that early context and aims? In what context and with what aims are the task and stimuli used currently? What light does the difference between the early context and aims and the current ones shed on the conceptual implications of the methods?
**Phase 3. Drawing consequences regarding comparability and validity**	*Comparability* To what extent are labels consistently associated with experimental contrasts? What labels are most appropriate for different experimental contrasts?
	*Validity* What methodological choices are invalid and need to be discarded? What methodological choices need to be improved and how? What avenues for further research suggest themselves given the limitations of current methodological approaches? What is the appropriate way to communicate the scope of findings, given the task and stimuli used?

### 2.1 Setting up an inventory of methodological choices

The first step in setting up a conceptual review is to build an inventory of the conceptually relevant methodological choices researchers have made in a particular subfield of CNS. These will always include task characteristics such as stimuli, instructions, required responses and control conditions. They may also include pre‐ or post‐scan questionnaires, where these are used in the analysis, and participant characteristics, where inclusion criteria for participants carry implications for the mental concept that is studied.^6^

All information can be presented in a table, or also in a formalized task ontology, about which more later. Presentation of methodological information in a table makes patterns immediately visible and differences between studies pop out. In the conceptual review I undertook of CNS of self‐reflection (van Stee, 2022, chapter 3), a clear pattern is that the majority of studies use personality trait adjectives as stimuli (Chiao et al., 2009; Craik et al., 1999; D'Argembeau et al., 2007, 2008, 2010; Fossati et al., 2003; Gutchess et al., 2007; Heatherton et al., 2006; Jenkins & Mitchell, 2011; W. M. Kelley et al., 2002; Kircher et al., 2000; Lieberman et al., 2004; Macrae et al., 2004; Moran et al., 2006; Ochsner et al., 2005; Schmitz et al., 2004; Zhu et al., 2007). Participants are instructed to judge whether or to what extent adjectives such as ‘ambitious’, ‘patient’ or ‘careless’ describe them. Only a few studies use other aspects of the self as stimuli, such as current mental states (e.g. ‘bored’) (e.g. Jenkins & Mitchell, 2011). One study includes not just words for participants to reflect on, but also modified pictures of their faces (Kircher et al., 2000). Participants are instructed to respond within 2 to 4 seconds, depending on the study.

Four studies immediately stand out, as they do not employ any stimuli and allow (much) longer response times, up to 120 seconds (D'Argembeau et al., 2005; Herwig et al., 2012; Johnson et al., 2006; Kjaer et al., 2002). In these studies, participants are required to self‐reflect rather freely, prompted by instructions such as “Try to think about your traits and attitudes, about your personality, in your relationship with others” for 100 seconds (D'Argembeau et al., 2005) or “reflect on who you are, how you are as a person” for 9.9 seconds (Herwig et al., 2012). After the experiment, researchers interview participants to check whether they have complied with the instructions.

Control tasks in CNS of self‐reflection mostly require participants to reflect on others rather than on the self, whether this other be the president (D'Argembeau et al., 2005; Jenkins & Mitchell, 2011; W. M. Kelley et al., 2002), the queen (Kjaer et al., 2002), the prime‐minister (Zhu et al., 2007), Einstein (Gutchess et al., 2007) or someone closer, such as an acquaintance, friend, sibling, romantic partner or mother (D'Argembeau et al., 2008; Kircher et al., 2000; Modinos et al., 2009; Ochsner et al., 2005; Schmitz et al., 2004; Zhu et al., 2007). Some studies use other control tasks, such as judging whether an adjective is written in uppercase or lowercase letters (Gutchess et al., 2007; W. M. Kelley et al., 2002; Zhu et al., 2007), or counting how many syllables it contains (Craik et al., 1999; Ochsner et al., 2005). Neural activity during the control task is subtracted from neural activity during the self‐reflection task, to determine what neural activity correlates specifically with self‐reflection.

The mental labels researchers have applied to their own studies vary. Besides ‘self‐reflection’, these are ‘self‐referential reflective activity’, ‘self‐referential processing’, ‘self‐processing’, ‘thinking about selves’, ‘self‐knowledge’, ‘reflective self‐awareness’, ‘metacognitive evaluation of the self’, ‘judgments of self’ and also simply ‘the self’.

### 2.2 Analysing conceptual implications of methodological choices

Methodological choices have implications for the mental concept that is being investigated. Theoretical assumptions about the concept are built into tasks. To be clear, neuroscientists need not explicitly hold these assumptions; researchers often choose the details of their tasks pragmatically. The time allowed for participants in the free self‐reflection studies is a nice example: 9.9 seconds seems like an odd amount of time to give to participants for self‐reflection unless you know that it equals the time needed to obtain five full brain scans through functional Magnetic Resonance Imaging (fMRI). Similarly, the studies allowing 100 or 120 seconds for self‐reflection need that time to accommodate the approximately 90 seconds it takes to acquire a full brain scan through Position Emission Tomography (PET), which is the technique used there (D'Argembeau et al., 2005; Kjaer et al., 2002). Yet all these pragmatic choices regarding tasks have conceptual implications, which in turn influence the comparability of studies.

Analysis of the conceptual implications of methodological choices can be facilitated in several ways. I envision these ways to run parallel to the ways in which philosophers draw out implicit assumptions underlying theoretical views and apply them to CNS' methods instead. The two main ones are investigating the history of a method and comparing different methods to each other.

First, one can investigate the context in which a task was originally developed. What aims did researchers have that made them develop the task in the way that they did? And how are these aims different from the context in which the task is currently used? In CNS of self‐reflection, the task that is used most often requires participants to judge quickly whether personality adjectives (‘stubborn’, ‘friendly’) apply to them or not. This task was originally developed in memory research, to demonstrate the so‐called ‘self‐reference memory effect’: people appear to remember adjectives better when they have processed them in reference to themselves as compared to processing that merely involves counting their syllables (see also Zahavi & Roepstorff, 2011). This explains why the first self‐reflection studies still involved a memory test at the end, and why they chose counting syllables as a control task. Knowing the task's history also elucidates why early studies often used labels like ‘self‐referential processing’ or ‘self‐referential reflective activity’ rather than ‘self‐reflection’. Furthermore, the history of the task explains why participants regularly only have 2 seconds to reflect on whether they are stubborn, whereas in ordinary language, the term ‘self‐reflection’ is not often used to refer to such quick judgments. Short presentation times of adjectives make sense when interested in people's ability to memorize those adjectives, but less so when interested in their capacity to self‐reflect.

The adjectives themselves have an entire history too. Current studies refer back to early studies in CNS of self‐reflection (mainly Craik et al., 1999), which refer to a study by Norman Anderson (1968) as the source of their adjectives. Anderson in turn selected adjectives from lists compiled by Allport and Odbert (1936). Allport and Odbert went through the entire Webster's Unabridged New English Dictionary of 1925 and jotted down 17,953 terms that could possibly describe personality. Anderson selected a subset using several criteria, one of which was to exclude adjectives that denote physical characteristics. ‘Strong‐minded’ thus made it onto his list, whereas ‘strong’ did not. That is, built into this stimulus set is the conceptual assumption that personality is of the mind and not of the body. Anderson obtained likeableness ratings for all selected adjectives and these are still used by researchers today to ensure that the valence of adjectives in the experimental and control conditions is counterbalanced. The likeableness ratings were obtained from 1960s university students, however, and it is questionable whether people from different socio‐demographic groups would be as positive about ‘intelligent’, ‘open‐minded’, ‘earnest’, ‘interesting’, ‘broad‐minded’, ‘well‐spoken’, ‘educated’, ‘clever’, ‘quick‐witted’ and ‘brilliant’, which are all in the top 50 of most likeable personality descriptors. The history of a task and its stimuli can thus provide insight into the take on self‐reflection that is embedded in it.

A second way to gain a clearer view of the implications of methodological choices is by comparing different tasks to each other, parallel to how philosophers compare different uses of concepts to each other. For CNS of self‐reflection, we can compare the most prevalent task of judging personality adjectives with studies that instruct participants to take some time to reflect freely on their personality. In the second case, when interviewed after the experiment, participants reported reflections such as “I am extrovert and talkative, while on the other hand I have sides only my friends know” (Kjaer et al., 2002, p. 1081). This stands in clear contrast to having to decide within two seconds whether one is extroverted, yes or no, as in the first task. Comparing the two tasks draws attention to the time that participants get and the content their reflections can possibly have. The second type of task corresponds more closely to ‘self‐reflection’ as the term is used in ordinary language, albeit in most studies only with respect to personality and not to other aspects of the self. The first task shares this focus on personality, but seems to allow for little reflection. Instead, I would argue, that task involves accessing a rough and ready representation of one's personality. This representation is rough and schematic, as yes/no answer possibilities do not allow for subtleties like being extroverted under certain circumstances but not others, being somewhat extroverted overall, or being less extroverted now than one was in the past. Instead of trying to gain a better understanding of themselves, which is what people mostly aim for through self‐reflection, participants need to quickly draw on an understanding of themselves that they already hold. Instead of constructing an updated version of their self‐understanding, they are thus passively accessing the self‐understanding that is already there. Accessing a personality schema in this way is different from reflecting on one's personality.

### 2.3 Drawing consequences regarding comparability and validity

Conceptual review can contribute to improving the validity of studies and the comparability of studies in a CNS subfield or also, between CNS and related disciplines. In other words, conceptual review can help overcome the issues outlined in section 1.

In order to overcome the comparability problem and facilitate meta‐analysis in CNS, the main question that needs addressing is: to what extent are labels consistently associated with experimental contrasts? In CNS of self‐reflection, roughly two types of tasks are employed. Yet at least ten different labels are used to refer to these two types of tasks. The four studies employing the free self‐reflection task receive three different labels: ‘self‐reflection’, ‘reflective self‐awareness’ and ‘self‐referential reflective activity’. The first of these, ‘self‐reflection’, is also often used to refer to studies employing the task of quickly accessing a rough and ready self‐schema. That is, different labels refer to the same task, and the same label refers to different tasks. Moving CNS of self‐reflection forward would involve aligning labels and tasks: ‘self‐reflection on personality’ seems an apt label for the stimulus‐free tasks, whereas ‘accessing one's personality schema’ seems an appropriate label for the personality adjective judgment task. These labels can also be used to group studies in review articles and databases, preventing the comparing of apples and oranges that arises when all studies are included as ‘self‐reflection’ studies.

The other version of the comparability problem is failing to compare apples with apples. As mentioned above, review articles in CNS of working memory fail to include studies labelled as ‘task switching’ studies, even when these employ the exact same experimental contrast as certain ‘working memory’ studies. As far as CNS of self‐reflection is concerned, I am not worried that review articles would fail to include studies because of their labels, as these labels all contain ‘self’ in some form and it is common to design search terms that cover variants. Across disciplinary boundaries, the problem does occur, however: psychologists investigating self‐schemas fail to find this CNS literature, even though they too make use of personality adjective judgment tasks, ever since the foundational study by Hazel Markus (1977).

Ultimately, what label is most appropriate is not a fact that can be discovered; it is a matter of analysis and argument. Likewise for comparability, where the million‐dollar question is: when are two types of tasks comparable *enough*? The criterion for comparability is clear, as it follows directly from CNS' aim to investigate the neural underpinnings of mental processes: studies should only be grouped together if their methods induce the same mental processes; if differences in methods lead to the involvement of different mental processes, they should be kept apart. Based on my conceptual review, I argue that the relevant difference within CNS of self‐reflection is between those two groups that involve self‐reflection on personality versus accessing a personality schema. I would hypothesize that the latter task involves declarative memory (or even semantic memory) processes to a greater extent than free self‐reflection does. Vice versa, self‐reflection on personality may rely on episodic memories in combination with reasoning processes to a greater extent than accessing a personality schema does.

As far as validity is concerned, a clear overview over tasks and control tasks allows one to weed out invalid tasks. The task of having to determine whether a modified picture of your face is you or a stranger is an invalid operationalization of self‐reflection I would argue. Lower‐level visual processing could potentially do the job, e.g. if large eyes indicate self, whereas small eyes indicate a stranger. Lack of validity is also something to watch out for when tasks that were developed in one context are taken into a different context. Using syllable counting or judging whether an adjective is written in uppercase or lowercase makes sense as a control condition when the aim is to study the self‐reference memory effect. I would argue that such control conditions make for an invalid self‐reflection task, however, as the experimental contrast captures reflection on people rather than self‐reflection specifically. Even control tasks like reflecting on the personalities of the president, the queen or Einstein may be confounded with familiarity, with neural activity underpinning reflection on familiar people, rather than self‐reflection specifically.

More generally, conceptual review brings unhelpful methodological choices to light, including choices that may reflect socio‐cultural or theoretical biases. It may be time to test the likableness of personality adjectives in people that are more diverse in terms of their socio‐economic background, gender and age than the 1960s students were. It may also be good to reappraise the exclusion of physical attributes from the personality adjective list: ‘strong’ may turn out to be a highly familiar description of personality and thereby an excellent stimulus in studies.

Although not wrong in itself, the focus on personality is a limitation to the scope of CNS of self‐reflection. Further research may want to include reflection on other aspects of the self. Conceptual review thus shows lacunae in research and thereby avenues for further research.

In general, then, conceptual review provides a clear view on the scope of the conclusions we can draw on the basis of neuroimaging studies, through providing a precise explication of the mental concept under study. This is important in itself, for the neuroscience community. It is also important when communicating with a wider audience, where findings are easily oversold as pertaining to a broader topic than they actually do, given the task they employed. Finally, it is important when communicating with researchers in adjacent disciplines. Integrating perspectives on self‐reflection or memory from different research fields only makes sense if researchers are not just using the same label, but mean the same thing by that label (Sullivan, 2016a, 2016b). Otherwise, we are again comparing apples and oranges. Methodological choices, particularly those pertaining to tasks, determine what has been studied exactly. Conceptual review helps to elucidate the precise mental concept that is embedded in those choices.

### 2.4 Conceptual review compared to validity checks in CNS

Good neuroscientists pay attention to the tasks they employ. They check whether a task is commonly used, whether it has face validity and, importantly, whether the control task does not introduce confounding factors. How exactly does conceptual review differ from such informal checks on validity that good neuroscientists already employ? Occasionally, researchers test the convergent validity of tasks, i.e. the extent to which two different tasks supposedly testing the same mental construct actually lead to converging results. What does a conceptual review have to offer over and above such approaches to validity checking?

At the very least, conceptual review involves an explication and systematization of informal validity checks that good neuroscientists already apply. Many neuroscientists would notice confounding factors in self‐reflection studies that rely on facial recognition or on the visual processing of adjectives as a control condition. Yet clearly, the original authors of these studies did not notice, nor did the researchers that reviewed their articles before publication. Conceptual review ensures that all neuroscientists have to pay attention to the validity of their tasks.

What is more, conceptual review is more systematic and digs deeper than even the best neuroscientists' informal checks can. It thus uncovers more relevant information. CNS of self‐reflection has been reviewed many times, in several cases even with an explicit eye to its conceptual side (Gillihan & Farah, 2005; Legrand & Ruby, 2009; Northoff et al., 2006, 2011; van der Meer et al., 2010; Vogeley & Gallagher, 2011; Zahavi & Roepstorff, 2011). Yet none of these reviews have explicated the conceptual implications of task choices for the precise mental concept under study. They have not noted the focus on personality rather than other aspects of the self. And none of these reviews have distinguished between the two types of tasks. Similarly, the assumption that personality is of the mind and not of the body that is embedded in current stimuli cannot be uncovered through informal checks only. Conceptual review brings out these embedded conceptual assumptions.

It is only when researchers recognize that they are using two different types of tasks to supposedly study the same mental process that they consider testing for convergent validity. In the case of CNS of self‐reflection, this has not happened yet; conceptual review brings out the need to do so. Tests of convergent validity can help decide whether two tasks tap into the same mental process or not. In working memory research for example, tests of convergent validity of the N‐back task with verbal span or digit span tasks show that the N‐back task correlates only weakly (Kane et al., 2007) or not at all (Redick & Lindsey, 2013). This finding plays a large role in arguments about the validity of the N‐back task as a task involving working memory. What tests for convergent validity cannot do, however, is ensure that either task is a valid operationalization of the concept of interest, as in, that either task truly elicits the mental process it is meant to elicit, including span tasks themselves. Relatedly, convergent validity does not in itself point to what mental label is appropriate for either task. Conceptual review contributes crucial information on those points.

## 3 IMPLEMENTING CONCEPTUAL REVIEW AND DISSEMINATING ITS RESULTS

So far, I have argued that meta‐analyses in cognitive neuroscience are often exposed to the comparability problem and that the comparability problem also hinders the integration of findings from adjacent disciplines. Tasks, and the experimental contrasts implemented through them, determine what has been studied exactly. Yet mental labels such as ‘working memory’ or ‘self‐reflection’ are applied in inconsistent ways to experimental contrasts: studies with the same label use different experimental contrasts and studies using the same contrast are labelled differently. What is more, tasks need not measure what they purport to be measuring in the first place, i.e. they may be invalid. I introduced conceptual review to address both the comparability problem and validity issues in cognitive neuroscience and illustrated the steps involved by doing a conceptual review on CNS of self‐reflection. I now turn to the practical implementation of conceptual review to give suggestions on how to facilitate its uptake by the CNS community. To be clear, these are suggestions only; they are possibilities that I can think of, but other ways to fruitfully implement conceptual review are bound to exist too.

A clear incentive exists to perform conceptual review: conceptual review is itself research worthy of publication, just like conceptual analysis in philosophy is, and ordinary review in cognitive neuroscience. The easiest way to disseminate a conceptual review to the relevant research community is via the usual routes: publish the conceptual review in neuroscience journals and present the review at conferences attended by cognitive neuroscientists investigating the mental concept at issue. They are the ones who stand to benefit from a precise analysis of the mental concepts operationalized by tasks and concrete suggestions as to what tasks to use, what mental label to apply to those tasks, and how to think of the relation between different tasks. To facilitate continued uptake by the research community, it would be practical to not just publish its results as an article but also enter the conceptual review's findings into an online cognitive ontology.

The basic informatics infrastructure for an online task ontology already exists. It is called the Cognitive Paradigm Ontology, or CogPO for short (cogpo.org; Turner & Laird, 2012). CogPO could be linked to Cognitive Atlas, as was in fact its original intention (Poldrack et al., 2011). Mental labels that result from conceptual review can be included in the Atlas. In turn, experimental contrasts resulting from task details can be linked to these updated mental labels through an ‘operationalizes’ relation. This will ensure that studies will be grouped according to the improved labels they have received. They will only be compared to each other and to related studies from different disciplines if these share the same label. Databases and the meta‐analyses that result from them will be organized accordingly.

Currently, Cognitive Atlas is a quiet place to visit, and CogPO is entirely asleep. The main problem plaguing them has been called the ‘data entry problem’. Ontologies such as Cognitive Atlas and CogPO currently rely on the input of many, yet researchers lack the incentive to participate in them (see Laird et al., 2005 for general suggestions on how to deal with this problem). In contrast, for the conceptual reviewer, a cognitive ontology would be a place to disseminate their conceptual research. This means the cognitive reviewer has an incentive to enter their analysis in the ontology and link it to the studies that they reviewed.

A further problem with wiki‐style ontologies such as Cognitive Atlas is that they have no safeguards to ensure the quality of entries. In principle, any researcher could give their fifty cents on what ‘working memory’ or ‘self‐reflection’ amounts to (and they do, see fn 5). Contributors tend to enter definitions for mental concepts based on the theory they espouse, but this in itself means, as explained above, that instead of resolving the comparability problem, researchers end up repeating it in the ontology. In contrast, a conceptual review is explicitly aimed at overcoming the comparability problem, through analysing task details. This results in precise labels and concrete suggestions, supported by argument, regarding which tasks are comparable enough to warrant the same label and which are different. Entries on the basis of conceptual review of task details will therefore be more valid and more comparable than current entries are.

Although it is possible in theory that careful review of task details for their conceptual implications will lead different conceptual reviewers to somewhat different conclusions, this does not undermine the relevance of conceptual review. First of all, we should not overestimate how often this will occur. In general, current groupings of studies will often be too coarse and decisions about comparability can be relatively straightforward. My proposal to divide the so‐called self‐reflection studies into two groups, one labelled ‘self‐reflection on personality’ and the other ‘accessing a personality schema’, is an example. In general, then, conceptual review is bound to greatly *reduce* disagreement compared to current practices, or rather, it will reduce inconsistencies in the ways mental labels and tasks are linked. What is more, a task ontology will contain all information that enables researchers to disagree *productively*, i.e. based on arguments that are themselves based on facts about employed tasks, rather than on whatever theory someone may espouse. A task ontology will also ensure that future researchers have access to all the relevant methodological information in this respect. All of this is an improvement over cognitive ontologies currently. All of this would improve meta‐analysis in cognitive neuroscience as well as the integration of neuroscientific findings with findings from adjacent disciplines.

Neuroscientists have good reason to look up a conceptual review when choosing a task for their own research: it safeguards the validity of their study and its comparability to other studies. However, to make sure that they actually do so, top‐down incentives would be helpful. Examples could include developing specific venues for the dissemination of conceptual reviews in journals and at conferences. Funding agencies and journal editors could require CNS studies to be based on tasks that have been vetted by conceptual review. We could draw inspiration from incentives put into place to promote the preregistration of analysis protocols in response to the replication crisis. Such incentives will only be put into place if the CNS community (or at least leading members of that community) believes it to be a good idea. This will only happen if we face the gravity of the consequences that result if we fail to do so.

CNS of self‐reflection has been around for 25 years by now. As mentioned in section 2.4, the field has been reviewed many times, yet none of those reviews note the two types of tasks that are used, or the overall focus on personality rather than other aspects of the self. None of the reviews result in more precise labels for the tasks used. If we look at trends over the years, we can see that the mental labels used in later years are no more uniform than in earlier years. Task use has become more uniform over the years though: the personality‐schema operationalization is virtually the only one used in later years (van Stee, 2022, chapter 3 and appendix B).

We could think of this as progress. CNS of self‐reflection seems to have converged on a technical definition for self‐reflection: in the context of neuroscience studies, self‐reflection simply means ‘quickly accessing schematic information about one's personality’. Researchers are unaware of this, sure, and they continue to use all sorts of labels, but most of those labels can be found by searching for ‘self*’. At least we have some uniformity in terms of the task.

Indeed, that much is true. But look at what we have lost.

First of all, we have lost societal relevance. For example, we will not learn anything from these studies about differences in neural correlates in people who are good versus bad at self‐reflection, for these studies are just not about what we ordinarily call self‐reflection. Participants have no time for examining their experience, drawing out information about themselves from experience and updating their self‐image accordingly. Instead, they can only quickly retrieve a schematic type of self‐image that they already hold.

Secondly, we are steering research in unfruitful directions. Researchers do not realize that the personality schema task does not capture self‐reflection. And so they are using it to investigate patient populations who are thought to suffer from impaired self‐reflection, e.g. patients with schizophrenia (e.g. Bedford et al., 2012; Holt et al., 2011). This is a waste of resources. What they are researching is neural differences between healthy and patient populations as they are quickly retrieving schematic information about their personality from memory. These researchers seem unaware that there is another task available that is more valid as an operationalization of self‐reflection.

Thirdly, we are failing to learn from these studies what we would be able to learn, were we to label them appropriately. We could potentially learn something from the personality schema studies about retrieving self‐schemas from memory and about neural correlates of that process. Currently, the research community is unaware of that.

Fourthly, we are failing to compare these studies to comparable ones, both within CNS and across disciplinary borders. Meta‐analyses on the neural correlates of memory processes pertaining to schematic information will not be included in this entire literature. Likewise, researchers from adjacent disciplines such as psychologists investigating self‐schemas do not know that this literature is of utter relevance to them. And just as we are failing to compare apples with apples, we are also comparing apples with oranges, when our meta‐analyses in CNS of self‐reflection include results obtained through two types of tasks that involve different mental processes.

Too much of researchers' time and energy and society's money are spent on research from which we are not learning what we could be learning, and on being misguided into unfruitful research directions because we are being sloppy about labelling our tasks. We need to be a lot more precise in our descriptions of the mental concepts induced by tasks. Where necessary, we could still learn better lessons from existing data if we redo meta‐analyses accordingly. Valid labelling of tasks and comparability across studies in this respect is exactly what conceptual review results in.

## 4 CONCLUSION

Meta‐analyses within cognitive neuroscience (CNS) and cross‐disciplinary integration of research findings rely on mental labels to decide which studies to compare. Currently, these labels are applied inconsistently to experimental contrasts. What is more, tasks need not be valid operationalizations of the mental labels that are attached to them. We need to pay more attention to task details of cognitive neuroscience studies to gain clarity regarding their comparability and validity.

Conceptual review is a systematic way to analyse tasks and their conceptual implications. It uses philosophical strategies to uncover the conceptual implications of task details. Its results facilitate precise communication of results and point out avenues for further research. It allows one to weed out invalid tasks and other unhelpful methodological choices. Importantly, the conceptual review's results elucidate the comparability of different studies. Conceptual review can stand on its own, yet combining it with a cognitive ontology may be beneficial: if cognitive ontologies were built up on the basis of the information conceptual review provides, they stand a much larger chance of succeeding at providing a standardized nomenclature for CNS than they do currently.

In sum, conceptual review addresses issues of comparability and validity in cognitive neuroscience. It thereby has the potential to improve the quality of meta‐analyses within CNS and of attempts at cross‐disciplinary integration, both of which are crucial for advancing neuroscience.

## CONFLICT OF INTEREST STATEMENT

The author declares no conflict of interest.

### PEER REVIEW

The peer review history for this article is available at https://www.webofscience.com/api/gateway/wos/peer-review/10.1111/ejn.16623.

## Data Availability

Sharing of neuroscientific data is not applicable as no neuroscientific data were created in this study.

## Footnotes

^2^
Cronbach and Meehl (1955) are widely cited as they are the *locus classicus* for the term ‘construct validity’. Their validity concept is more technical than the broad one I use here, however, and less applicable to CNS. Specifically, their validity concept focuses on test scores and their interpretation. Yet tasks in CNS experiments regularly simply induce a mental process, without measuring performance, and therefore without resulting in the test scores necessary for Cronbach and Meehl's validity concept to apply.

^3^
The page for ‘working memory’ contains a definition that runs on for 53 words, the first of which are “active maintenance and flexible updating of goal/task relevant information (items, goals, strategies, etc.).” Next to information on working memory's “asserted relationships to other concepts”, the page also contains a list of “tasks that are asserted to measure working memory”: 24 tasks in total.

^4^
E.g. “The Sternberg Item Recognition Task is a working memory task requiring participants to first encode a set of digits (encode‐phase) and then to maintain them on‐line in WM while responding to each of the probe digits that follow by indicating whether or not it was a member of the memorized set (probe‐phase).” The Sternberg task page contains a list of three mental concepts that the task “has been asserted to measure.”

^5^
E.g. ‘working memory maintenance’, ‘active maintenance’, and ‘maintenance’ are all registered as parts of working memory and they point to the same mental construct, but are defined differently. In this way, 16 mental concepts are asserted to be a part of working memory, without anybody believing that working memory has 16 component processes.

^6^
For example, a conceptual review of CNS of love showed that participants in CNS experiments on romantic love were recruited with flyers asking ‘Are you truly, madly, deeply in love?’, thereby narrowing romantic love to the peak experience of infatuation (van Stee, 2022, chapter 2).
